# Maternal dopamine exposure provides offspring starvation resistance in *Daphnia*

**DOI:** 10.1002/ece3.8785

**Published:** 2022-04-01

**Authors:** Semona Issa, Safa Chaabani, Alexandros G. Asimakopoulos, Veerle L. B. Jaspers, Sigurd Einum

**Affiliations:** ^1^ 8018 Department of Biology Centre for Biodiversity Dynamics Norwegian University of Science and Technology Trondheim Norway; ^2^ 8018 Norwegian Agriculture Agency Oslo Norway; ^3^ 8018 Department of Chemistry Norwegian University of Science and Technology Trondheim Norway; ^4^ 8018 Environmental Toxicology Department of Biology Norwegian University of Science and Technology Trondheim Norway

**Keywords:** fitness, life history, maternal effects, phenotypic plasticity, reaction norms

## Abstract

The neurotransmitter dopamine has been shown to play an important role in modulating behavioral, morphological, and life history responses to food abundance. However, costs of expressing high dopamine levels remain poorly studied and are essential for understanding the evolution of the dopamine system. Negative maternal effects on offspring size from enhanced maternal dopamine levels have previously been documented in *Daphnia*. Here, we tested whether this translates into fitness costs in terms of lower starvation resistance in offspring. We exposed *Daphnia magna* mothers to aqueous dopamine (2.3 or 0 mg/L for the control) at two food levels (*ad libitum* vs. 30% *ad libitum*) and recorded a range of maternal life history traits. The longevity of their offspring was then quantified in the absence of food. In both control and dopamine treatments, mothers that experienced restricted food ration had lower somatic growth rates and higher age at maturation. Maternal food restriction also resulted in production of larger offspring that had a superior starvation resistance compared to *ad libitum* groups. However, although dopamine exposed mothers produced smaller offspring than controls at restricted food ration, these smaller offspring survived longer under starvation. Hence, maternal dopamine exposure provided an improved offspring starvation resistance. We discuss the relative importance of proximate and ultimate causes for why *D*. *magna* may not evolve toward higher endogenous dopamine levels despite the fitness benefits this appears to have.

## Graphical abstract

Costs of expressing high dopamine levels remain poorly studied and are essential for understanding the evolution of the dopamine system. Here, we tested whether enhanced maternal dopamine levels translate into offspring fitness costs, but found an opposite effect, with increased longevity under starvation for their offspring. Thus, it remains unclear what constrains the evolution of higher endogenous dopamine levels.

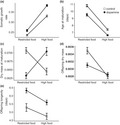

## 1 INTRODUCTION

Organisms living in spatially and temporally heterogeneous environments can be expected to express phenotypic plasticity (e.g., Lázaro‐Nogal et al., 2015), the propensity of an organism to change its phenotype in response to changes in the environment (West‐Eberhard, 2003). Under natural selection, adaptive phenotypic plasticity evolves such that the resulting reaction norm gives higher fitness across the changing environment (Lande, 2009). Phenotypic plasticity can also be transferred from mother to offspring such that the maternal response to the environment induces changes to the offspring reaction norm (Uller, 2008). Maternal effects can then impact offspring fitness and ultimately population dynamics (Benton et al., 2008). Hence, expression of adaptive reaction norms is essential to maintain high fitness as well as population viability in heterogeneous environments. This is especially the case for reaction norms that are expressed in response to food abundance, as changes in these can have strong effects on different components of life history (Boggs, 2009). In short‐lived species, for example, resource allocation to somatic maintenance (including survival) increases at the cost of growth and reproduction when food is limited (Lynch, 1989; Martínez‐Jerónimo et al., 1994). Resulting changes in maternal resource allocation to offspring can ultimately influence offspring survival and reproduction (Enserink et al., 1995; Hafer et al., 2011; Saastamoinen et al., 2013).

At the physiological level, the neurotransmitter dopamine plays an important role as mediator of trait responses to food (see Barron et al., 2010, for a review on dopamine‐mediated behavioural and morphological responses to food across taxa). Issa et al. (2020) showed that in addition to influencing morphological and behavioral traits, dopamine can regulate life history responses to food abundance. Specifically, in the zooplankton species *Daphnia magna*, exposure to dopamine caused life history reaction norms (age at maturation and fecundity) to change in a way that resulted in higher population growth rates (calculated from maternal life history traits) when food was limited (Issa et al., 2020). This happened without any apparent fitness costs at high food abundance. These observations raise the question of why endogenous dopamine levels do not evolve toward higher values. Issa et al. (2020) suggested this may be due to costs of high dopamine levels being paid by the offspring generation, which was not quantified in their study. Negative maternal effects on offspring size from dopamine treatments were detected. A smaller offspring size may have detrimental effects on offspring survival since body size in *Daphnia* is positively associated with filtering rates (Porter et al., 1983) and offspring survival under food limitation (Gliwicz & Guisande, 1992). Because offspring survival is a crucial component of maternal fitness, investigation of costs to offspring from enhanced maternal dopamine levels could give better insight into the selective forces shaping the evolution of the dopamine system.

In this study, we experimentally tested for effects of maternal dopamine exposure on offspring fitness. We exposed one generation (F_0_) of *D*. *magna* to dopamine at high vs. restricted food ration and starved the offspring (F_1_) to measure their starvation resistance. Based on the previous findings of Issa et al. (2020), we predicted changes to the slopes of the life history reaction norms under dopamine exposure that result in faster somatic growth and smaller offspring when food is limited. Moreover, we hypothesized that the smaller offspring originating from dopamine‐exposed mothers would experience reduced survival under starvation.

## 2 MATERIALS AND METHODS

### 2.1 Study organisms

As previously outlined in Issa et al. (2020), ephippia (i.e., the capsule containing resting eggs) resulting from sexual reproduction of *D*. *magna* were collected in November 2014, in a pond at Værøy Island (1.0 ha, 67.687°N 12.672°E), northern Norway. Ephippial eggs were hatched in the laboratory and propagated clonally. For the current experiment, juveniles of a single clone (clone 49) of *D*. *magna* were asexually propagated for 10 successive generations prior to use. A maximum of 30 individuals of *D*. *magna* were cultured in 2.5‐L aquaria at 20°C in a modified “Aachener Daphnien Medium” (ADaM) (Klüttgen et al., 1994, SeO2 concentration reduced by 50%), under long photoperiods (16 h L: 8 h D) using white fluorescent lamps. The medium was exchanged weekly and the animals were fed three times a week with Shellfish Diet 1800^®^ (Reed mariculture Inc.) at a final concentration of 3.2 × 10^5^ cells/ml.

### 2.2 Experimental design

**FIGURE 1:**
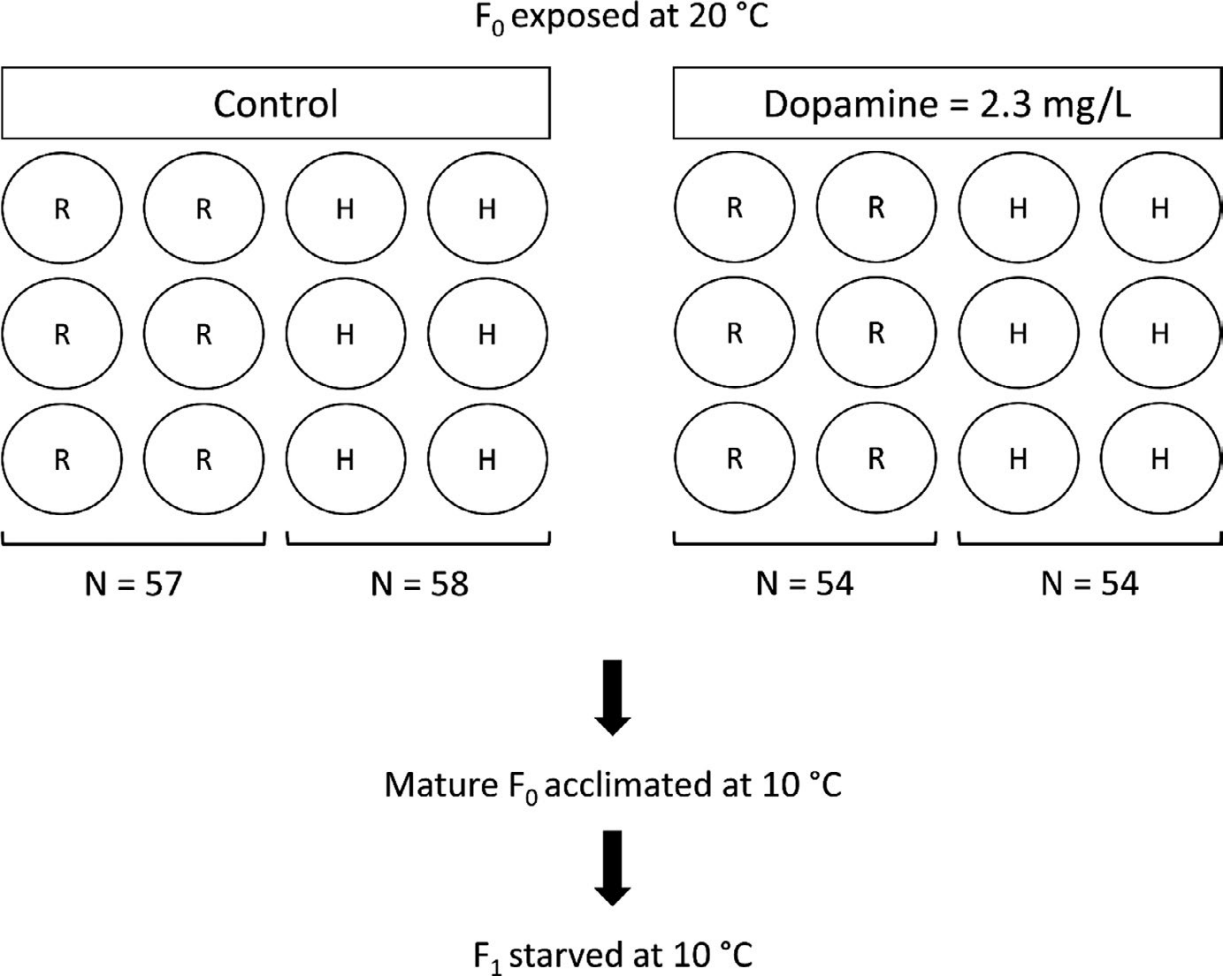
Schematic diagram of the experimental design. The F_0_ generation was kept at restricted (R) and high (H) food ration for both control and dopamine treatments (2 x 2 design) from birth until maturity, with six replicate beakers per treatment combination and with each replicate initially containing 10 individuals. Due to the occurrence of males in the F_0_ generation, total sample sizes, given as N, varied among treatments. Individuals in the F_1_ generation were kept without food in separate wells and were not exposed to dopamine

For the F_0_ generation, a similar experimental design as in Issa et al. (2020) was used, corresponding to a full factorial design with control, dopamine, and two food rations (high vs. restricted), with six 500‐ml replicate beakers (non‐aerated borosilicate beakers, Fisherbrand) for each of the four combinations (Figure 1). Ten neonates (<24 h old) were introduced into each beaker and kept at 20°C under long photoperiods (16 h L: 8 h D) until maturation. The exposure concentration of dopamine (2.3 mg dopamine hydrochloride/L) was chosen for successfully inducing changes in *D*. *magna* growth (Issa et al., 2020; Weiss et al., 2015). An exposure protocol as previously outlined in Issa et al. (2020) was followed. Specifically, dopamine hydrochloride (Sigma‐Aldrich) was first dissolved in 100 ml ultrapure water before dilution in ADaM to the desired exposure concentration. Controls containing only ADaM medium were performed parallel to the exposure replicates. The medium was renewed in all replicates (*N* = 24) three times a week, and the animals were fed at each renewal event with Shellfish Diet 1800^®^ at a final concentration of 2.88 × 10^5^ cells/ml (*ad lib* at 20°C) for the high food ration and 8.6 × 10^4^ cells/ml (30% *ad lib* at 20°C) for the restricted food ration.

All replicates were checked daily for mature individuals. In *Daphnia*, maturity can be determined by visual inspection of the brood chamber (the dorsal part of the animal) through the transparent carapace, into which fully developed eggs are oviposited. Mature individuals were removed from the beakers, photographed for size measurements (see Section 2.3 below), and kept individually in 2‐ml non‐aerated wells containing ADaM at 10°C until they had released their neonates. Seventeen of the 240 individuals in the beakers were found to be males and were thus not transferred (Figure 1). To prevent released neonates from obtaining food, no food was added to the wells. The wells were checked daily and once neonates had been released (i.e., <24 h old, F_1_ generation), two individuals were randomly sampled from each well, photographed for size measurements (see Section 2.3 below), and transferred individually to new 2‐ml non‐aerated wells containing ADaM (no food) at 10°C. The medium was not exchanged, and individuals were checked daily to record mortality. The low exposure temperature was chosen to slow down metabolism and increase longevity under starvation, thus making it more likely to accurately quantify any variation in offspring longevity under the chosen frequency of observations.

### 2.3 Sampling procedure and measurements of life history traits

Immediately prior to dopamine exposure on day 0 of the experiment, a random sample of 30 F_0_ neonates were photographed for body length measurements (mm, measured from the upper margin of the eye to the junction of the carapace and spine, using ImageJ v1.52a, National Institutes of Health, Bethesda, MD). Individual measurements of body length at first reproduction for the F_0_ generation (after releasing their first clutch of eggs) and at birth for the F_1_ generation were measured in the same way. All body length measurements (mm) were converted into dry mass (mg) using the equation by Yashchenko et al. (2016): Dry mass = 0.00535 × Body length^2.72^. The somatic growth rate in the F_0_ generation was calculated for each individual as: $\mathrm{Somatic}\;\mathrm{growth}\;\mathrm{rate}=\frac{\ln\left({\mathrm{dry}\;\mathrm{mass}}_{\mathrm{end}}\right)‐\ln\left({\mathrm{dry}\;\mathrm{mass}}_{\mathrm{start}}\right)}{\mathrm{Duration}}$, where dry mass _start_ is the average dry mass (in mg) at the neonatal stage (day 0), dry mass _end_ is the individual dry mass (in mg) at maturation, and duration is the number of days between the two stages.

As in Issa et al. (2020), conductivity (WTW LF 330 conductivity meter), pH (WTW pH 340i), and dissolved oxygen (WTW Multi 3410 multiprobe meter) were measured. This was done on two separate occasions throughout the experiment (Table 1), after medium renewal, in the new exposure solutions and ADaM medium used for the controls (*N* = 4; two samples collected in total from each of the dopamine and control treatments). Simultaneously, the new exposure solutions and ADaM medium were sampled for dopamine analysis (*N* = 4; two samples collected in total from each of the dopamine and control treatments).

**TABLE 1:** Water quality measurements from study on life history responses to food ration and dopamine exposure in *Daphnia magna*

Sampling event	Treatment	Dopamine (mg/L)	Conductivity (mS/cm)	Dissolved oxygen (mg/L)	pH
1	Dopamine	0.052	1.64	8.83	7.95
1	Control	0	1.653	8.93	8.02
2	Dopamine	0.056	1.527	8.97	7.98
2	Control	0	1.541	9.02	8.01

The samples were stored at −80°C for a maximum of 5 months after collection (during closure of the labs due to the Covid‐19 outbreak) prior to dopamine analysis. Two complementary sample preparation protocols were used: (1) dilute‐and‐shoot; and (2) liquid–liquid extraction. Subsequent analysis was performed by ultra‐performance liquid chromatography coupled to a triple quadrupole mass analyzer (UPLC‐MS/MS). This analysis was done as described in Issa et al. (2020), where single reaction monitoring (SRM) chromatograms were monitored for dopamine. Typical SRM chromatograms for dopamine in the analyzed samples are depicted in Appendix S1. Over the course of the experiment, pH, conductivity, and dissolved oxygen were within the recommended range for testing of chemicals in *D*. *magna*, according to OECD guidelines (OECD, 2012). The conductivity remained at 1.6 mS/cm, mean dissolved oxygen at 8.9 mg/L, and pH at 8.0 across treatments, whereas measured average concentrations of dopamine were ca. 2% of their nominal concentrations (Table 1). Much lower than expected concentrations of this compound were likely caused by degradation during unforeseen long‐term storage due to the Covid‐19 outbreak.

### 2.4 Statistical analyses

All statistical analyses and graphic illustrations were performed in R v. 3.6.3 (R Core Team, 2020). We tested whether the slopes of the reaction norms of the measured life history traits in response to food abundance differed among dopamine treatments (control vs. dopamine exposure). To assess this for dry mass at maturation and somatic growth rate, we used linear mixed effects models (LME, implemented using the *lme* functions in the package *nlme*, Pinheiro et al., 2020), with fixed effects of treatment and food ration and the interaction between these, and with beaker ID as a random effect. For offspring dry mass, LME models were fitted with treatment, food ration, and their interaction as fixed predictor variables, and maternal ID nested in beaker as a random predictor variable. Finally, we tested the effects of treatment, food ration and their interaction on age at maturation and offspring longevity (both being probability distributions), using Poisson generalized linear mixed effects (GLMM, implemented using the *glmer* function in the package *lme4*, Bates et al., 2015). For these latter analyses, beaker ID and maternal ID nested in beaker ID were included as random effects, respectively. For LME models, the VarIdent command from the *nlme* package was used to allow residual variance to differ among treatments, food ration, and the two‐way interaction between these (Pinheiro & Bates, 2000). Using a chi‐square test of the residual deviance and degrees of freedom, GLMM models were found not to be overdispersed, and their Pearson and deviance residuals were found to satisfy model assumptions. For all analyses, the appropriate random structure was first established by comparing the Akaike's information criterion with correction for small sample size (AIC_c_) of models that included all fixed factors but differed in their random effects structure. Once the appropriate random structure was determined, fits of alternative models with different fixed effects structures were compared, again using AIC_c_.

## 3 RESULTS

**FIGURE 2:**
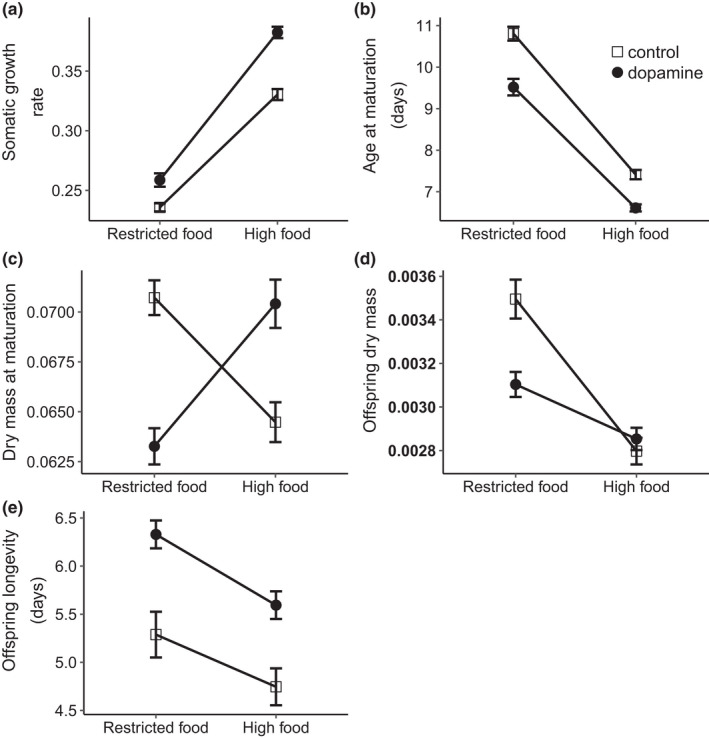
Effect of food ration on F_0_ and F_1_ traits in *D*.* magna* in the dopamine and control treatments. (a) Somatic growth rate, (b) age at maturation (days), (c) dry mass at maturation (mg), (d) offspring dry mass (mg) and (e) offspring longevity (days). Error bars give 1SE

All life history traits were influenced by both food ration and dopamine treatments (Table 2). Most of them responded in the same direction to a change in food ration for both control and dopamine treatments. Specifically, somatic growth rate increased with higher food ration, whereas age at maturation, offspring dry mass, and offspring longevity decreased (Figure 2, Table 3). For dry mass at maturation, there was a strong interaction between food ration and dopamine treatment (Table 2). While dry mass at maturation decreased with increasing food ration in the control treatment, the opposite pattern was observed in the dopamine treatment (Figure 2c, Table 3).

**TABLE 2:** Model comparison of candidate models for testing effects of treatment (control vs. dopamine) and food ration (high vs. restricted) on somatic growth rate, age at maturation, dry mass at maturation, offspring dry mass, and offspring longevity in *D*.* magna*. The least complex model within two ΔAIC_c_ is given in bold. vI refers to the varIdent function

Response variable	Model	*K*	AIC_c_	∆AIC_c_	wAIC_c_
Somatic growth rate					
Fixed	**Food: Treatment**	7	−870.40	0.00	0.76
	Food + Treatment	6	−868.10	2.30	0.24
	Food	5	−850.80	19.61	0.00
	Treatment	5	−811.40	59.05	0.00
	~1	4	−810.90	59.47	0.00
Random	**vI (Treatment) + (1|Beaker)**	7	−837.66	0.00	0.64
	vI (Food: Treatment) + (1|Beaker)	9	−836.46	1.20	0.35
	(1|Beaker)	6	−829.30	8.36	0.01
	vI (Food) + (1|Beaker)	8	−827.41	8.81	0.00
Age at maturation	**Food + Treatment**	3	925.51	0.00	0.69
	Food: Treatment	4	927.58	2.07	0.25
	Food	2	930.52	4.99	0.06
	Treatment	2	968.65	43.14	0.00
	~1	1	968.89	43.37	0.00
Dry mass at maturation					
Fixed	**Food: Treatment**	6	−1545.60	0.00	1.00
	~1	3	−1533.10	12.52	0.00
	Treatment	4	−1531.20	14.42	0.00
	Food	4	−1531.10	14.54	0.00
	Food + Treatment	5	−1529.20	16.46	0.00
Random	vI (Food:Treatment) + (1|Beaker)	9	−1501.62	0.00	0.34
	vI (Treatment) + (1|Beaker)	7	−1501.13	0.49	0.27
	vI (Food) + (1|Beaker)	7	−1501.08	0.54	0.26
	**(1 | Beaker)**	6	−1499.81	1.81	0.14
Offspring dry mass					
Fixed	**Food: Treatment**	7	−3361.70	0.00	0.63
	Food + Treatment	6	−3359.54	2.16	0.21
	Food	5	−3358.98	2.73	0.16
	~ 1	4	−3349.57	12.13	0.01
	Treatment	5	−3349.31	12.39	0.01
Random	**vI (Treatment) + (1|Beaker/Maternal)**	7	−3295.35	0.00	0.50
	vI (Food: Treatment) + (1|Beaker/Maternal)	9	−3295.20	0.15	0.46
	(1 | Beaker/Maternal)	6	−3289.77	5.58	0.03
	vI (Food) + (1 | Beaker/Maternal)	7	−3287.78	7.57	0.01
Offspring longevity	**Food + Treatment**	3	1065.75	0.00	0.60
	Food: Treatment	4	1067.83	2.08	0.21
	Treatment	2	1068.23	2.48	0.17
	Food	2	1073.39	7.64	0.01
	~1	1	1074.54	8.79	0.01

**TABLE 3:** Parameter estimates from the best models (Table 2) describing the response of life history traits to food ration and dopamine exposure in *Daphnia magna*

Response variable	Final model	Parameter	Estimate ± SE
Somatic growth rate	Food: Treatment + vI (Treatment) + (1|Beaker)	Intercept	0.24 ± 0.006
		Dopamine treatment	0.02 ± 0.01
		High food	0.09 ± 0.009
		High food: Dopamine treatment	0.03 ± 0.01
Age at maturation (days)	Food + Treatment + (1|Beaker)	Intercept	2.38 ± 0.04
		Dopamine treatment	−0.12 ± 0.05
		High food	−0.37 ± 0.05
Dry mass at maturation (mg)	Food: Treatment + (1|Beaker)	Intercept	0.07 ± 0.001
		Dopamine treatment	−0.007 ± 0.002
		High food	−0.006 ± 0.002
		High food: Dopamine treatment	0.01 ± 0.003
Offspring dry mass (mg)	Food: Treatment + vI (Treatment) + (1|Beaker/Maternal)	Intercept	0.003 ± 0.0001
		Dopamine treatment	−0.0004 ± 0.0001
		High food	−0.0007 ± 0.0001
		High food: Dopamine treatment	0.0004 ± 0.0002
Offspring longevity (days)	Food + Treatment + (1|Beaker/Maternal)	Intercept	1.67 ± 0.04
		Dopamine treatment	0.17 ± 0.05
		High food	−0.12 ± 0.05

The steepness of the food ration reaction norms depended on dopamine treatment for somatic growth rate and offspring dry mass (Table 2). Specifically, dopamine exposure increased somatic growth rate, but this was more pronounced at high food ration (Table 3), causing the reaction norm to be steeper for the dopamine exposed group compared to the control (Figure 2a). For offspring dry mass, treatment had no effect at high food ration, whereas exposure to dopamine reduced offspring dry mass at restricted food ration (Table 3). Thus, for this trait, the reaction norm was steepest for the control group (Figure 2d). In contrast, exposure to dopamine lowered age at maturation to an equal extent across food rations (Figure 2b, Table 2). Similarly, the effect of dopamine treatment on offspring longevity did not depend on the maternal food ration (Table 2). Rather, exposure to dopamine increased offspring longevity across food rations (Figure 2e).

## 4 DISCUSSION

In a previous study, Issa et al. (2020) investigated the role of dopamine in shaping life history responses to food abundance in *D*. *magna*. This was done both through aqueous exposure to dopamine and to the antidepressant bupropion, a dopamine reuptake inhibitor. Both treatments led to higher population growth rates (calculated from maternal life history traits) when food was restricted, without any apparent costs to fitness. The higher dopamine exposure resulted, however, in smaller offspring, which could potentially perform worse particularly when facing restrictions in food availability (Gliwicz, 1990). Since offspring survival is a crucial component of maternal fitness, this may represent a fitness cost of higher maternal dopamine levels. In the current study, we tested this by exposing F_0_ *D*. *magna* to aqueous dopamine and quantifying life history responses of mothers (F_0_) to food abundance as well as the starvation resistance of their offspring (F_1_).

Similar to the findings of Issa et al. (2020), offspring size was larger in mothers that had experienced restricted food abundance, a pattern commonly observed in *Daphnia* (Garbutt & Little, 2014; Glazier, 1992), as well as in other organisms (Reznick et al., 1996; Vijendravarma et al., 2010). This response was in the same direction for both dopamine and control treatments but was considerably steeper for the control treatment. The latter arose as a consequence of dopamine exposure causing a reduction in offspring size when mothers experienced restricted food ration, but not when mothers received *ad lib* food. In general, a larger investment in offspring size when food is limited is expected to boost offspring survival and fitness (Gorbi et al., 2011; Tessier & Consolatti, 1989). In support of this, we found that the larger offspring of mothers having experienced restricted food ration survived better under starvation across treatments. Mothers that experience low food abundance tend to produce offspring with a larger maternal lipid reserve, which can increase offspring starvation resistance (Tessier et al., 1983). In contrast to our hypothesis, however, maternal exposure to dopamine did not come at a cost to offspring longevity. Surprisingly, offspring in the dopamine treatment survived longer than controls across food rations. This was true even for the offspring from mothers that had experienced restricted food rations, where offspring size was smaller in the dopamine treatment than in the controls. Hence, enhanced maternal dopamine levels increased rather than decreased offspring survival.

Unlike the effects of dopamine exposure on offspring longevity, its effects on maternal life history reaction norms were overall as predicted based on the findings of Issa et al. (2020). Specifically, at restricted food ration, somatic growth rate decreased, and maturation was delayed. However, dopamine exposure resulted in faster growth and earlier age at maturation across food rations compared to the control. Higher dopamine levels have been shown to promote cell proliferation and/or increase cell volume, explaining the positive effect of dopamine exposure on somatic growth rate (Huet & Franquinet, 1981; Weiss et al., 2015). As in Issa et al. (2020), a smaller adult size was observed at restricted food ration in the dopamine treatment, with potential costs to adult survival and reproduction (Cleuvers et al., 1997; Lampert, 2001), as body size in *Daphnia* positively correlates with the ability to satisfy metabolic requirements at low food levels. Contrastingly, at high food ration, dopamine exposure increased adult size, and hence *Daphnia* competitiveness (Brooks & Dodson, 1965), compared to the control. The significant effects of dopamine treatment on adult size and somatic growth rate (and consequently age at maturation) at high food ration were surprising, given that treatment effects were expected to occur at restricted food ration only (Issa et al., 2020). There were, however, some differences in the setup between the current study and the study by Issa et al. (2020) that may potentially explain this: (1) in the present study, we performed exposure in groups, thus allowing for interactions between individuals, whereas Issa et al. (2020) exposed animals individually; (2) the present study used a different clone from Issa et al. (2020). Previous research shows that antidepressant effects on life history traits of *Daphnia* species can vary between clones, if they differ in their growth and reproductive performance (Campos et al., 2012). Thus, the generality of our results, both with respect to offspring fitness costs and maternal life history reaction norms, is somewhat limited by the use of a single clone. Yet, although the exact patterns may depend on clonal identity and experimental protocols, the results of the present study support the overall conclusion of Issa et al. (2020) that important life history responses to food abundance are shaped by the dopamine system.

The observed increase in *Daphnia* growth and advanced timing of reproduction from elevated dopamine levels may also be interpreted as a stress response. Indeed, evidence shows that fast species (i.e., short‐lived species, producing many offspring early in life), such as *Daphnia*, respond to stressful environments by accelerating their life cycle (Rochet et al., 2000; Trippel, 1995). Higher dopamine levels may stress individuals through dopamine oxidation. Elevated intracellular dopamine levels can promote the intracellular oxidation of dopamine, by increasing the production of reactive quinones and free radicals for a given rate of oxidation (Miyazaki & Asanuma, 2008; Sun et al., 2018). In addition, increased extracellular dopamine can overload the antioxidant capacity in the extracellular space, causing dopamine oxidation (Blesa et al., 2015; LaVoie & Hastings, 1999; Miyazaki & Asanuma, 2008). In the case of limited investment in antioxidant defense, dopamine oxidation can then lead to oxidative stress, which can potentially reduce longevity (Ishii et al., 1998; Moskovitz et al., 2001). On the other hand, increased investment in antioxidant defense may lower investment in immune defense (Takahashi et al., 2017), ultimately increasing the susceptibility of individuals to diseases and parasites. A higher investment in antioxidant defense may also lower investment in reproduction (Speakman & Garratt, 2013). There may also be ecological costs of rapid growth associated with enhanced dopamine levels due to biotic interactions. One such cost may be higher predation risk from faster growth (Urban, 2007), as individuals increase growth rates by increasing feeding rates, which in turn can lead to increased predator exposure (Lankford et al., 2001; Stoks et al., 2005). Thus, whether through oxidative stress, or increased investment in antioxidant defense, dopamine oxidation may pose a proximate restriction for why *Daphnia* do not produce more endogenous dopamine. This in addition to more ultimate restrictions in the form of reduced survival, from oxidative stress, reduced immunity or increased predation risk, as well as lowered reproduction may be important to explore in order to understand the evolution of the dopamine system.

In summary, maternal dopamine exposure boosted *Daphnia* offspring survival in addition to accelerating its life cycle. Altogether, these results emphasize the important role of dopamine as a regulator of life history responses to food abundance but leave open the question of why *D*. *magna* do not evolve toward higher endogenous dopamine levels despite the apparent fitness benefits. A potential proximate cause may be that higher dopamine levels promote dopamine oxidation. More ultimate causes involve the potential costs of dopamine oxidation to reproduction and survival, as well as ecological costs of rapid growth due to biotic interactions (predation). Hence, further understanding of the evolution of the dopamine signaling system may require a combined investigation of ultimate and proximate causes.

## CONFLICT OF INTEREST

The authors have no conflict of interest to declare.

## AUTHOR CONTRIBUTIONS

**Semona Issa:** Conceptualization (equal); investigation (lead); writing – original draft (lead); writing – review and editing (lead). **Safa Chaabani:** Investigation (supporting); writing – review and editing (supporting). **Alexandros G. Asimakopoulos:** Investigation (supporting); methodology (supporting); writing – review and editing (supporting). **Veerle L. B. Jaspers:** Methodology (equal); writing – review and editing (supporting). **Sigurd Einum:** Conceptualization (equal); methodology (equal); resources (supporting); supervision (supporting); writing – review & editing (supporting).

## Supporting information

Supplementary Material

## Data Availability

All data used for this work are available at https://doi.org/10.5061/dryad.63xsj3v4d.
