# Supplementary material for: Maternal dopamine exposure provides offspring starvation resistance in Daphnia
Source: Ecol Evol. 2022 Apr 1;12(4):e8785. doi: 10.1002/ece3.8785 (PMC8975792; doi:10.1002/ece3.8785)
Supplement: Supplementary file 1 — Supplementary Material [file ECE3-12-e8785-s001.docx]

**Appendix 1**

Dopamine was confirmed with the highest degree of certainty since 4 SRM transitions were monitored instead of a single transition (Fig. A1). Each SRM transition denotes a characteristic fragmentation pathway of the target molecule. We considered a positive finding in the samples when all four SRMs demonstrated a positive signal in the expected retention time for dopamine. The control samples did not present any signal in any of the SRM transitions, denoting non-detectable concentrations.


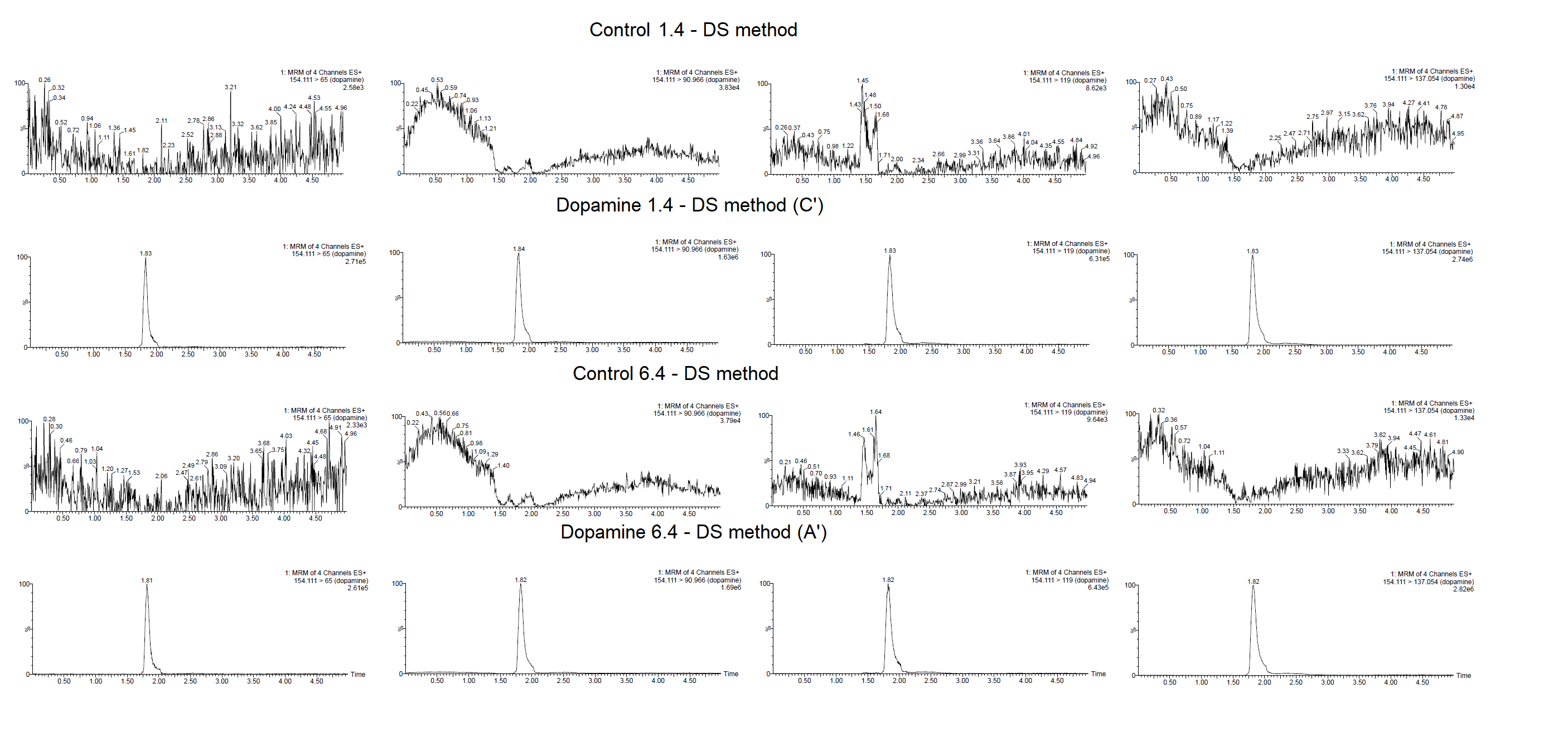


**Fig. A1.** Single Reaction Monitoring (SRM) chromatograms for dopamine in the four samples analysed; transitions monitored were: 154.1 > 137.0, 154.1 > 90.9, 154.1 > 119.0 and 154.1 > 65.0 m/z. Sampling dates were 1 April 2020 (denoted by 1.4) and 6 April 2020 (denoted by 6.4
